# Passatempo Virus, a Vaccinia Virus Strain, Brazil

**DOI:** 10.3201/eid1112.050773

**Published:** 2005-12

**Authors:** Juliana A. Leite, Betânia P. Drumond, Giliane S. Trindade, Zélia I.P. Lobato, Flávio G. da Fonseca, João R. dos Santos, Marieta C. Madureira, Maria I.M.C. Guedes, Jaqueline M.S. Ferreira, Cláudio A. Bonjardim, Paulo C.P. Ferreira, Erna G. Kroon

**Affiliations:** * Universidade Federal de Minas Gerais, Belo Horizonte, Minas Gerais, Brazil; † Centro de Pesquisas René Rachou–Fundação Instituto Oswaldo Cruz, Belo Horizonte, Minas Gerais, Brazil; ‡ Instituto Mineiro de Agropecuária, Belo Horizonte, Minas Gerais, Brazil

**Keywords:** zoonotic poxvirus, poxvirus outbreak, *Orthopoxvirus*, Passatempo virus, Araçatuba virus, Cantagalo virus, Vaccinia virus, *ha* gene, *vgf* gene, *tk* gene, dispatch

## Abstract

Passatempo virus was isolated during a zoonotic outbreak. Biologic features and molecular characterization of hemagglutinin, thymidine kinase, and vaccinia growth factor genes suggested a vaccinia virus infection, which strengthens the idea of the reemergence and circulation of vaccinia virus in Brazil. Molecular polymorphisms indicated that Passatempo virus is a different isolate.

Since 1999, an increasing number of exanthemous outbreaks affecting dairy cattle and cow milkers in Brazil have been reported (*1**–**3*). These outbreaks were related to poxvirus infections, which resulted in economic losses to farmers and affected the health of humans and animals. Here we report a vaccinia virus (VACV) outbreak that emerged in March 2003 in the town of Passa-Tempo, Minas Gerais State, Brazil.

## The Study

The outbreak area is characterized by small rural properties with diverse crops, pasturelands, and surrounding fragments of Atlantic Forest. Its climate is tropical, with a relatively severe dry season, generally from April to September (*4*).

**Figure 1:**
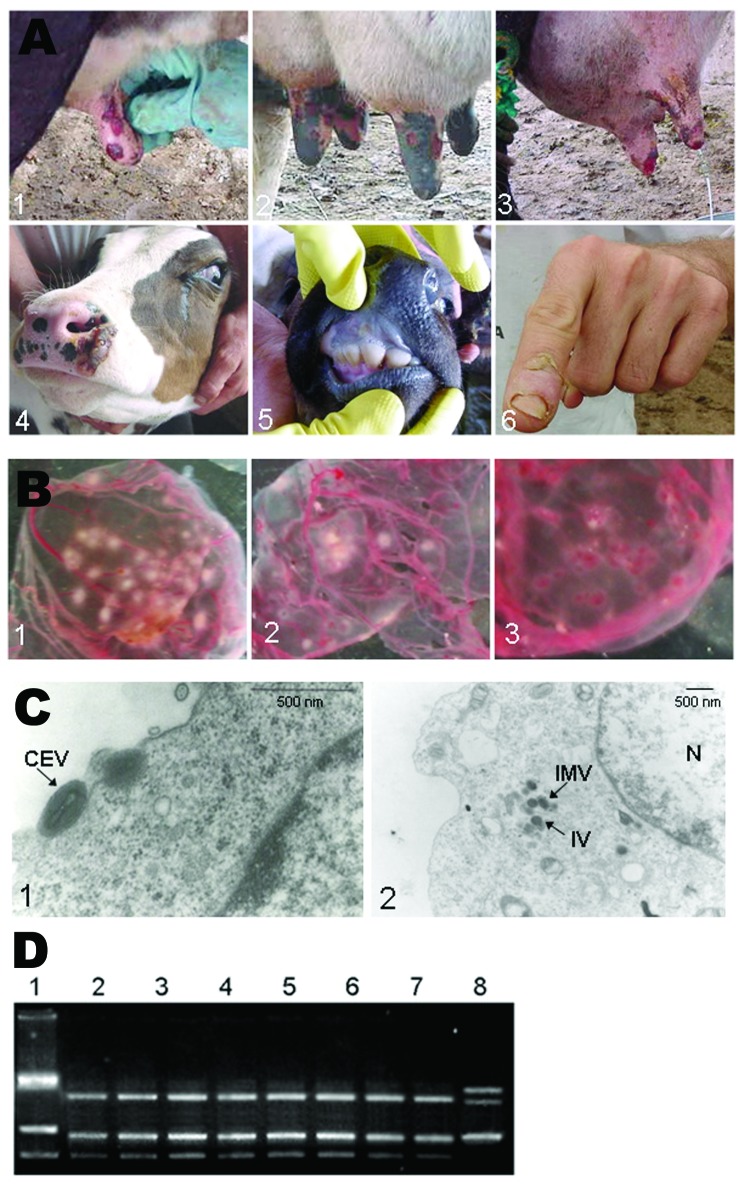
Lesions caused by Passatempo virus infection. Panels 1 and 2, ulcerative lesions on cows' teats; 3, mastitis caused by bacterial secondary infection; 4 and 5, lesion on calves' muzzle and oral mucosa; 6, lesions of dairy farm milker.

All dairy farms were similar, consisting of a main house with corrals and pasture fields generally with unsophisticated infrastructure. All milking was manually performed by milkers, typically without strict aseptic measures, which could have contributed to the spread of the virus among the herd and milkers. Cows exhibited lesions on teats and udders that resembled the clinical features observed during other Brazilian VACV outbreaks (*1*). Initial acute lesions were associated with a roseolar erythema with localized edema that led to the formation of vesicles. The vesicles rapidly progressed to papules and pustules, which subsequently ruptured and suppurated. Typically, a thick dark scab followed, but the formation of large areas of ulceration was also common. The course of infection lasted from 3 to 4 weeks. Different stages of lesions were present, ranging from papules to vesicles, pustules, and crusts (Figure 1). Moreover, because of secondary infections, some cows had mastitis (Figure 1). Calves became infected, showing lesions on oral mucosa and muzzles (Figure 1). Several infected milkers reported lesions on their hands, which were apparently transmitted by unprotected contact with sick cattle (Figure 1). In addition, infected persons reported severe headache, backache, lymphadenopathy, and high fever.

**Figure A1:**
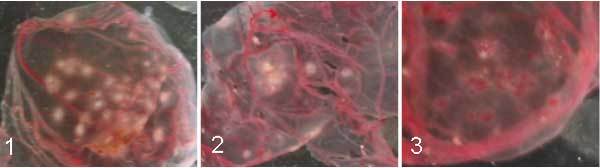
Pocks morphology in chorioallantoic membranes. 1 and 2, whitish and opaque pocks could be observed after an incubation period of 72 hours with the Passatempo and vaccinia virus Western Reserve (WR), respectively, on chorioallantoic membrane of 10-day-old embryonated pathogen-free eggs; 3, cowpox virus Brighton Red (BR) formed red hemorrhagic lesions.

For virus isolation, crusts were collected from 5 cows and 1 calf, macerated, and added to the chorioallantoic membrane of embryonated eggs (*2*). The whitish pockmarks produced on chorioallantoic membranes resembled VACV pocks, differing from the red hemorrhagic ones produced by cowpox virus (CPXV) (Figure A1). Blood from affected animals was collected for neutralization assays (*5*). Serologic cross-reactivity of antibodies to VACV–Western Reserve (WR) strain was detected in all samples, and titers of these serum samples were >640 U/mL (data not shown).

**Figure A2:**
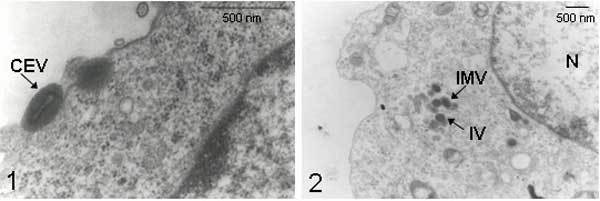
Electron micrographs of cells infected with Passatempo virus. Vero cells were infected with Passatempo virus at a multiplicity of infection of 5 and fixed by OsO4, 24 hours after inoculation. 1, typical orthopoxvirus "brick-shaped" morphologic pattern, with biconcave core and outer membranes were observed in cell-associated enveloped virus (CEV); 2, intracelullar mature virions (IMV) were visualized in the cytoplasm of infected cells within virosomes with the presence of spheric immature virus (IV). No A-type inclusion body (ATI) was visualized in infected cells. N, nucleus. Bars, 500 nm.

**Figure A3:**
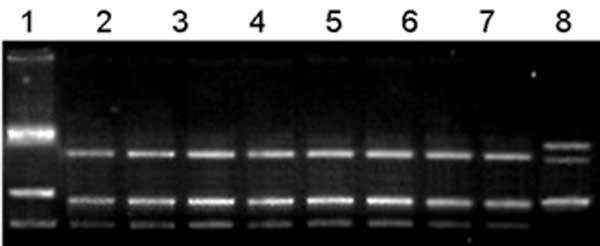
Restriction fragment length polymorphism analysis of the ati gene of viral isolates. ati fragments were amplified by polymerase chain reaction by using primers based on the cowpox virus ati gene nucleotide sequence, followed by XbaI digestion. The amplified DNA was fractionated by electrophoresis on a 1% agarose gel and stained with ethidium bromide. Lanes: 1, vaccinia virus WR; 2, isolate-cow1; 3, isolate-cow2; 4, isolate-cow3; 5, isolate-cow4; 6, isolate-cow5; 7, isolate-calf; 8, cowpox virus BR.

Transmission electron microscopy of isolates (*6*) showed a morphologic pattern typical of orthopoxviruses (Figure A2). No A-type inclusion body (ATI) was seen, reinforcing the conclusion that this virus was likely not a CPXV, but a VACV. Viral DNAs were extracted (*6*) and used as template for *ati* gene restriction fragment length polymorphism (RFLP) analysis (*7*). The *ati* RFLP patterns of all isolates were identical to those of Araçatuba virus (ARAV) (*1*) and other VACV strains previously isolated in our laboratory (unpub. data); they were similar to those of VACV-WR and completely different from those of CPXV-Brighton Red (BR) (Figure A3). Since all isolates showed the same *ati* RFLP pattern, one was cloned, purified, titrated (*1**,**6*), and named Passatempo virus (PSTV).

To better identify this etiologic agent, *ha*, *tk*, and *vgf* genes were amplified by polymerase chain reaction with Taq polymerase (Promega, Madison, WI, USA) (*6**,**8**,**9*). Amplicons were cloned into pGEM-T vector (Promega). Three clones were sequenced 3 times in both orientations by the dideoxy method, using M13 universal primers and ET Dynamic Terminator for MegaBACE (GE Healthcare, Fairfield, CT, USA). The nucleotide (nt) sequences of *ha*, *tk*, and *vgf* were assembled by using the CAP3 Sequence Assembling Program (*10*) and deposited in GenBank under accession numbers DQ070848, DQ085461, and DQ085462, respectively. The sequences and inferred amino acid sequences were aligned with those of orthopoxviruses by using the ClustalW 1.6 program (*11*).

**Figure A4:**
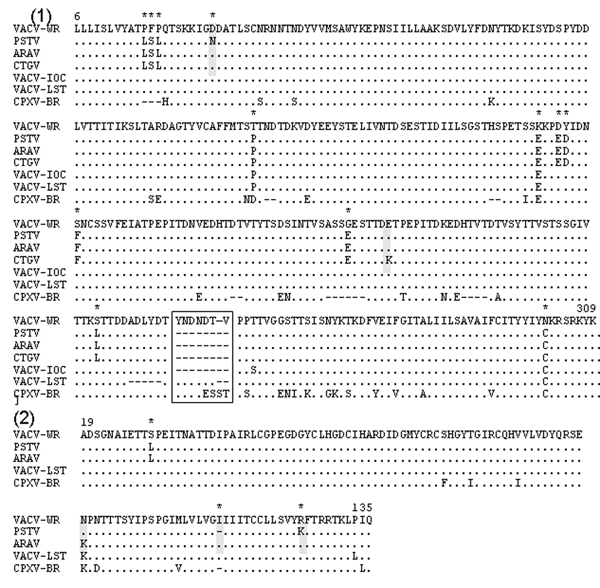
Alignment of Passatempo virus (PSTV) hemagluttinin antigen (HA) and vaccinia grown factor (VGF) amino acid inferred sequences with other orthopoxvirus sequences. 1, HA sequence of PSTV (DQ070848) was compared to the region corresponding to amino acids 6 to 309 of vaccinia virus (VACV-WR, AY243312) HA sequence and to HA sequences of Araçatuba virus (ARAV, AY523994); Cantagalo virus (CTGV, AF229247); vaccinia virus IOC (VACV-IOC, AF229248); vaccinia virus Lister (VAVC-LST, AF375124) and cowpox virus Brighton Red (BR) (CPXV-BR, AF482758). 2– The VGF sequence of PSTV (DQ085462) was compared to the region corresponding to amino acids 19 to 135 of vaccinia virus WR (VACV-WR, AY243312) and to the same region of Araçatuba virus (ARAV, AF503170), vaccinia virus Lister (VACV-LST, AY678276); and cowpox virus BR (CPXV-BR, AF482758). The sequences were obtained from GenBank and aligned by using the default parameters of CLUSTAL W and hand-edited to enhance biologically significant regions. The amino acid positions are shown according to the VACV-WR HA and VGF protein sequences. (.) indicates identity of amino acids, (-) indicates deletions, and (*) indicates the PSTV amino acid substitutions. The box shows the conserved deletion observed in the HA sequence of the Brazilian VACVs. Amino acids substitution found amongst PSTV and other Brazilian VACVs are shown in gray boxes.

PSTV *ha* gene sequence was compared to those of ARAV, Cantagalo virus (CTGV) (*1**,**2*), VACV-WR, CPXV-BR, VACV Instituto Oswaldo Cruz (VACV-IOC), and VACV Lister (VACV-LST). VACV-IOC and VACV-LST are vaccine strains used in the Brazilian smallpox eradication program (*2**,**6*). The PSTV *ha* gene sequences presented the same 18-nt deletion found in ARAV, CTGV, and VACV-IOC and shared more similarities to ARAV and CTGV homologous sequences. Additionally, 8 amino acid substitutions were unique to PSTV, ARAV, and CTGV. Since this characteristic was not observed in the vaccine strains, an independent origin is suggested. Moreover, PSTV HA differs from that of ARAV and CTGV by 1 and 2 amino acid substitutions, respectively (Figure A4). The percentage of identity between *ha*, *tk*, and *vgf* nucleotide sequences and inferred amino acid sequences of PSTV with CPXV-BR and other VACV strains are presented in the Table. For the *tk* gene that is highly conserved among VACV, the PSTV nucleotide sequence had 100% identity to ARAV, VACV-LST, and VACV-WR homologous sequences. Additionally, PSTV *vgf* gene had a 3-nt deletion, corresponding to nt 7,669–7,671 of VACV-WR, causing the loss of 1 isoleucine in a stretch of 4 found in the ARAV and VACV-WR VGF sequences (Figure A4). PSTV VGF also exhibited 2 amino acid substitutions when compared to ARAV VGF sequences.

**Table:** Passatempo virus (PSTV), Araçatuba virus (ARAV), Cantagalo virus (CTGV), Vaccinia virus IOC (VACV-IOC), vaccinia virus Lister (VACV-LST), vaccinia virus Western Reserve (VACV-WR), and cowpox virus Brighton Red (CPXV-BR) ha, tk, and vgf genes and amino acid sequences*†

PSTV	Identity among homologous sequences (%)					
	ARAV	CTGV	VACV-IOC	VACV-LST	VACV-WR	CPXV-BR
Genes						
*ha*	99.9	99.8	98.7	97.4	96.3	84.0
*tk*	100.0	–*	–*	99.8	100.0	98.0
*vgf*	98.6	–*	–*	98.0	98.6	95.7
Amino acids						
HA	99.7	99.3	96.6	95.0	94.0	79.9
TK	100.0	–*	–*	100.0	100.0	98.2
VGF	97.4	–*	–*	100.0	97.4	92.2

*CTGV and VACV-IOC *tk* and *vgf* nucleotide and amino acid sequences are not available in the GenBank database.

†HA, hemagglutinin; TK, thymidine kinase; VGF, vaccinia growth factor.

**Figure 2:**
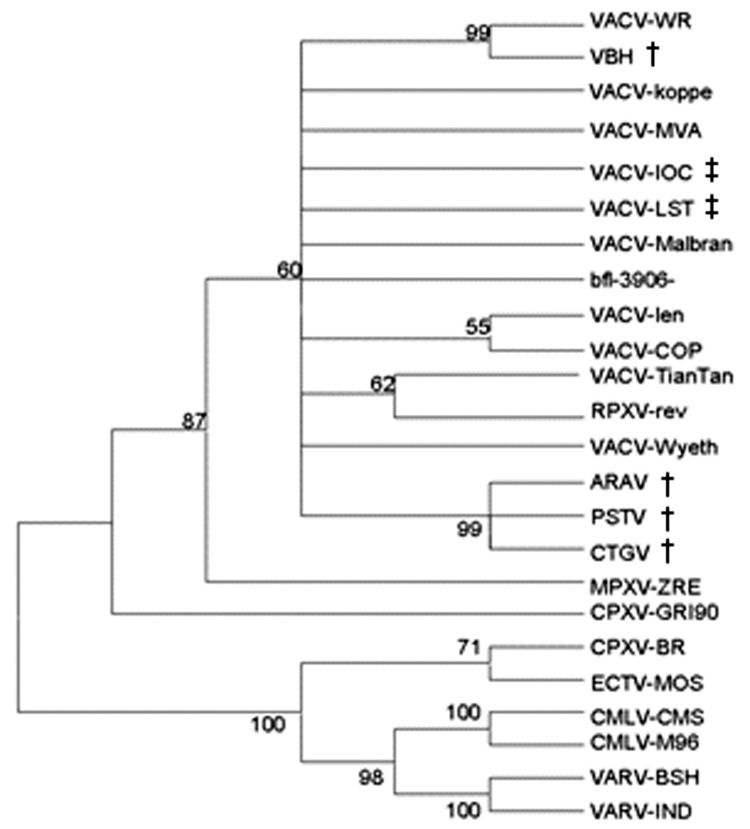
Consensus bootstrap phylogenetic tree based on the nucleotide sequence of Orthopoxvirus ha gene. The tree was constructed by the neighbor-joining method using the Tamura-Nei model of nucleotide substitutions implemented in MEGA3. The tree was midpoint-rooted, 1,000 bootstrap replicates were performed, and values >50% are shown. Nucleotide sequences were obtained from GenBank under accession numbers: PSTV (DQ070848), ARAV (AY523994), CTGV (AF229247), VACV-Wyeth (VVZ99051), VACV-TianTan (U25662), VBH (AY542799), VACV-WR (AY243312), VACV-Koppe (AF375122), VACV-MVA (U94848), VACV-IOC (AF229248), VACV-LST (AF375124), VACV-len (AF375123), VACV-COP (M35027), bfl-3906 (AF375077), VACV-Malbran (AY146624), RPXV-rev (AF375118), CPXV-GRI90 (CVZ9904), CPXV-BR (AF482758), ECTV-MOS (AF012825), CMLV-CMS (AY009089), CMLV-M96 (AF438165), VARV-BSH (L22579), VARV-IND (X69198), MPXV-ZRE (AF380138). (†) indicates Brazilian VACV isolates and (‡) indicates Brazilian vaccine samples.

The alignments were used to construct phylogenetic trees by the neighbor-joining method using the Tamura Nei model implemented in MEGA3 (*12*). Trees were rooted at midpoint, and 1,000 bootstrap replications were performed. A *tk* and *vgf* genes concatenated phylogenetic tree was constructed by placing PSTV together with VACV strains (data not shown). Regarding *ha* sequences, PSTV was clustered to ARAV and CTGV (Figure 2).

## Conclusions

The phylogenetic tree analysis suggested a strong phylogenetic relationship between PSTV and other Brazilian VACV strains. However, the *vgf* and *ha* gene analysis of PSTV, ARAV, and CTGV indicated that genetic heterogeneity exists among these viruses, which suggests that the *ha* gene deletion found in PSTV, ARAV, CTGV, and VACV-IOC could be a signature of New World or Brazilian VACV strains.

Additionally, that RFLP analysis showed a pattern identical with other Brazilian strains, similar to VACV-WR and different from CPXV, suggests that a cladogenesis event may have occurred. This conclusion is feasible considering that these viruses could be circulating in the wild since smallpox vaccination or even before, going back to the colonization of South America, when cattle and other animals were brought to the New World without quarantine or inspection. The VACV variants buffalopox and rabbitpox have originated from VACV subspeciation (*13*).

That humans were also infected and that these persons were all milkers, phenomena that had been observed during other Brazilian VACV outbreaks, points to an occupational zoonosis. Although parapoxvirus infection has been placed in the category of occupational zoonosis, to our knowledge no other orthopoxviruses have been reported to cause an occupational hazard. Economic losses are also a matter of concern. In addition to the reduction in milk production, extra veterinary costs are due to the usual occurrence of secondary infections on cows' teats leading to mastitis. The reduction in milk production is a concern because Brazil is a major milk exporter. Therefore, the spread of these viruses could severely impact the country's economy. In this regard, the clinical features, widespread dissemination, and epidemiology of the etiologic agent of these outbreaks must be understood.

**Figure 3:**
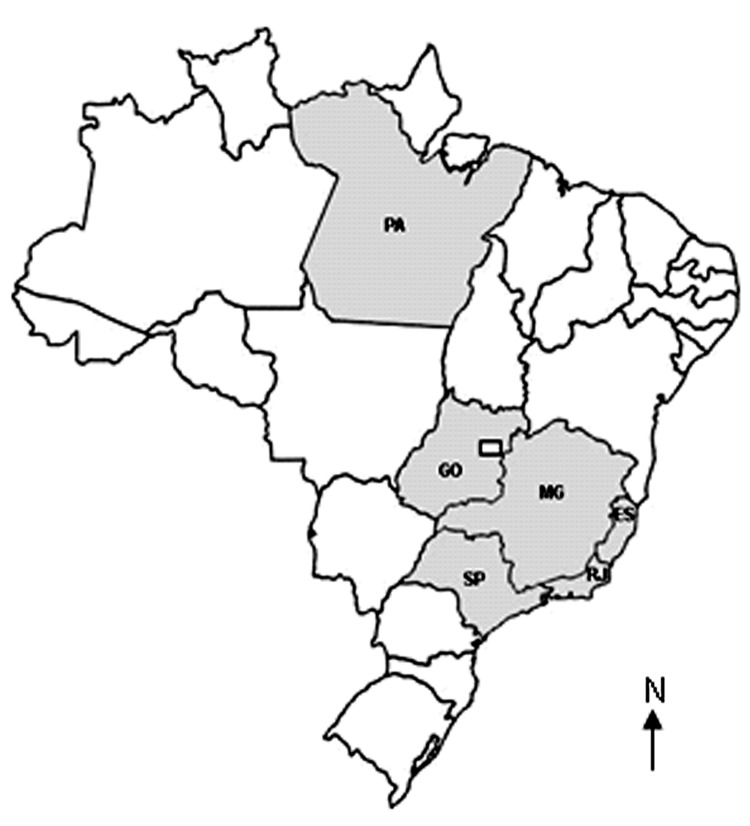
Brazilian states where vaccinia viruses were isolated. ES, Espírito Santo State: Espírito Santo isolates in 2004 (unpublished); GO, Goiás State: Goiás isolates after 2001 (*3*); MG, Minas Gerais State: Belo Horizonte virus in 1993 (*15*), Minas Gerais isolates after 2001 (*3*), Passatempo virus in 2003; PA, Pará State: BeAn 58058 virus in 1963 (*6*); RJ, Rio de Janeiro State: Cantagalo virus in 1999 (*2*); SP , São Paulo State: SPAn232 virus in 1979 (*14*), Araçatuba virus in 1999 (*1*), São Paulo isolates after 2001 (*3*).

Since 1963, all Brazilian orthopoxvirus isolates have been characterized as VACV strains (*3**,**6**,**14**,**15*). The growing geographic distribution of these outbreaks (Figure 3) indicates that these viruses may be emerging as zoonotic pathogens of cattle. This fact is especially important because a growing human population has no vaccine-derived immunity to smallpox or other orthopoxviruses. This situation could create an opportunity for these viruses to disseminate in Brazil. In addition, the isolation of another VACV strain strengthens the hypothesis that VACV is circulating in the New World and that these viruses seem to be endemic of this region.
